# Pseudochondritis in Leprosy

**DOI:** 10.4269/ajtmh.2010.10-0324

**Published:** 2010-12-06

**Authors:** Christophe Deligny, Serge Arfi

**Affiliations:** Service de Médecine Interne 5D, Centre Hospitalier Universitaire de Fort de France, Fort de France, Martinique, French West Indies

**Figure 1.**
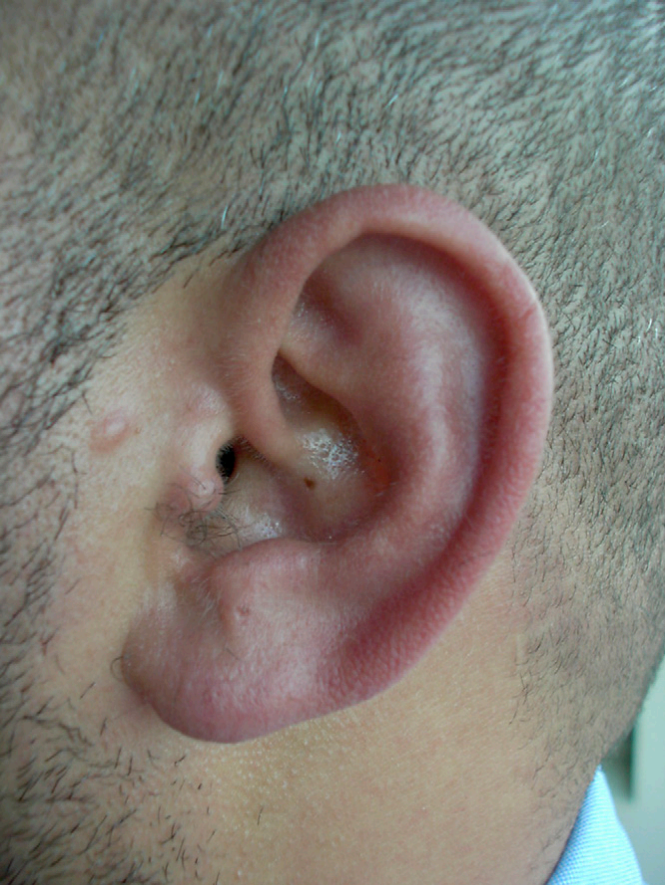
Pseudochondritis in an Afro-Carribean man with leprosy. This figure appears in color at www.ajtmh.org.

A healthy 32-year-old Afro-Caribbean man was referred to our hospital with suspected systemic vasculitis and chondritis because of symmetrical polyarthritis, recurrent erythematous lesions of the trunk and the face previously considered to be urticarian, and extremities swelling with distal symmetrical glove and stock sensory loss over a two-year period. He did not receive any antibiotics and corticosteroids. We observed bilateral ear pseudochondritis (Figure 1) with skin involvement of the lobule and limited tenderness, which excluded true chondritis that involved only cartilaginous areas, as seen in relapsing polychondritis, Wegener's granulomatosis, or mouth and genital ulcers with inflamed cartilage (MAGIC) syndrome.

True chondritis usually shows tenderness. Differential diagnosis of unilateral or bilateral pseudochondritis included infections, trauma, insect bite, tophaceous gout, radiotherapy, allergy, burn, or chilblain. Slight maculopapular lesions on the face compatible with leonine facies, papular erythematous geographic plaque-like lesions with two punch-out areas, presence of acid-fast bacilli in skin biospy specimens, and mucosal excretions enable diagnosis of lepromatous leprosy.

Pseudochondritis is rarely reported in leprosy, and today is seen only in the lepromatous type.^1^ Ulceration, megalobule, and nodulation over the pinna may be seen.^2^ The patient was treated with rifampin, ofloxacine, and disulone. He showed rapid improvement at a one-year follow-up, but disease recurrence occurred one year later.
